# Influence of Niobium on the Beginning of the Plastic Flow of Material during Cold Deformation

**DOI:** 10.1155/2013/723725

**Published:** 2013-12-19

**Authors:** Stoja Rešković, Ivan Jandrlić

**Affiliations:** University of Zagreb, Faculty of Metallurgy, Aleja Narodnih Heroja 3, 44103 Sisak, Croatia

## Abstract

Investigations were conducted on low-carbon steel and the steel with same chemical composition with addition of microalloying element niobium. While tensile testing was carried out, the thermographic measurement was tacking place simultaneously. A specific behavior of niobium microalloyed steel was noticed. Test results have shown that, in the elastic deformation region, thermoelastic effect occurs, which is more pronounced in niobium microalloyed steel. Start of plastic flow in steel which is not microalloyed with niobium begins later in comparison to the microalloyed steel, and it is conducted so that, at the point of maximum stress, deformation zone is formed within which stresses grow. In steel microalloyed with niobium after proportionality limit, comes the occurrence of the localized increase in temperature and the occurrence of Lüders band, which propagate along the sample forming a deformation zone.

## 1. Introduction

Niobium microalloyed steel is low carbon steel with the addition of niobium up to 0.06%. During thermomechanical treatment, niobium forms very small, stable, and hard niobium carbides, nitrides, and carbonitrides of various compositions. During thermo mechanical treatment, they are excreted in the form of densely arranged carbonitride series and represent a strong obstacles to movement of dislocations during deformation [1, 2]. Accumulation of dislocations around the series of precipitates in the deformed grain creates the subboundaries and by process of recrystallization, the reduction of grain size is achieved [2]. In this way, a fine grained structure and significantly improved properties are achieved compared to the same low-carbon steel without the addition microalloying element niobium.

During the static tensile testing in some steels, comes the occurrence of localized plastic deformation after reaching the yield point. This phenomenon is the explained by rheological behavior of materials during the cold deformation. Investigations conducted in recent years the occurrence of localized deformation associated with Lüders bands [3–6]. Lüders bands occur in some low carbon steels, which have a clearly defined upper and lower yield point. Niobium microalloyed steel has also a clearly defined upper and lower yield point, and at certain conditions, shows a rheological behavior at the beginning of plastic deformation.

There are different interpretations of reasons for appearance of Lüders bands and deformation mechanisms that take place at the same time. It was considered that the main reason for occurrence of Lüders bands is pinning of dislocations on the dissolved C and N atoms and their compounds which form the so-called Cottrell's atmospheres, so dislocations accumulate itself on them [6–8]. These mechanisms are not sufficiently clarified.

Recently, new methods have been started to be used for investigation of plastic flow of metals such as thermography, differential image correlation, and magnetic Barkhausen method. They allow the determination of the different parameters of deformations that are beginning to be used to create the mathematical models [3, 6, 8, 9]. At plastic deformation, a certain part of the work of plastic deformation turns into heat energy. A change in temperature of the sample during the static tensile tests is measured by a thermovision method. The temperature on the surface of the sample infrared camera converts in to the image on the camera screen, called a thermogram. By the subsequent analysis of the obtained thermograms, quantitative values of temperature distribution in the examined sample can be obtained. In this way, it is possible to visualize the inhomogeneous deformation during the progress of Lüders bands.

The purpose of this paper is to investigate the beginning of plastic flow of niobium microalloyed steel during static tensile testing. Using the thermal vision method, it will be observed the formation and propagation of Lüders bands. The study will show the influence of niobium precipitates on the formation of the deformation zone.

## 2. Experimental Work

Research was conducted on the hot-rolled strips from low carbon steel and the same steel microalloyed with niobium, whose chemical composition is shown in Table 1.

Samples for testing were taken from the hot-rolled strips with thickness of 3 mm in the rolling direction. Dimensions of tested samples were as follows: the gage length is 45 mm, width is 20 mm, and thickness is 3 mm. Before testing, the samples were sprayed with black matte coating, which gave the uniform emissivity factor of 0.95. Static tension test was performed on Zwick 50 kN testing machine at a constant rate of stretching 15 mm/min.

During the static tensile testing, load-elongation curves were continuously recorded, and the surface of the samples was recorded with infrared camera. Infrared camera used was VarioCAM M82910, which has the ability to detect changes in the temperature of 80 mK. The camera was calibrated to room temperature and set at an angle of 15° in relation to the tested sample. The set parameters within the cameras software that was used during the tests are: frequency of recording of 50 frames per second, an emissivity factor of 0.95, distance of camera from the object of 0.75 m, and 240% of zoom was used. Conditions in the laboratory during the testing were 21.3°C and relative humidity was 66%. The following analysis of the results of thermovision tests was done by IRBIS-3 professional software.

## 3. Results and Discussion

As the thickness of the sample was only 3 mm, it was assumed that the measured changes in the temperature of surface are equal to the change in the internal temperature of samples. Previous studies on the influence of type of coatings and emissivity factors onto the measured results suggest that its quality is acceptable [10].

**Figure 1:**
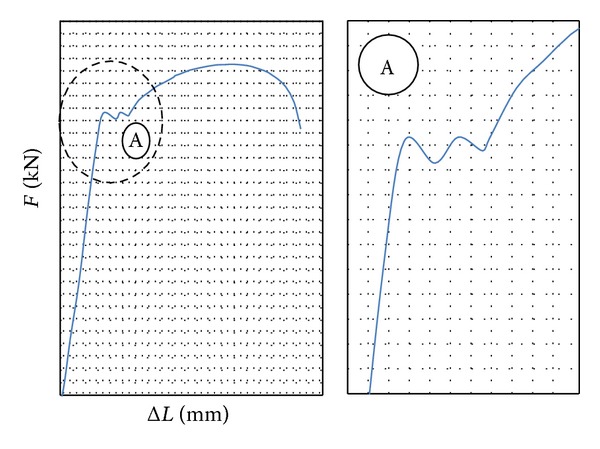
Relationship between loading force and elongation at static tensile test.

Recorded load-elongation curve, Figure 1, obtained by static tensile tests, shows that tested niobium microalloyed steel has distinct upper and lower yield point. After reaching the yield point, its complex rheological behavior can be seen, Figure 1, detail A. The appearance of oscillations on the increase of loading force before the start of plastic flow of material was not recorded in the same low-carbon steel without the addition of niobium [2].

**Figure 2:**
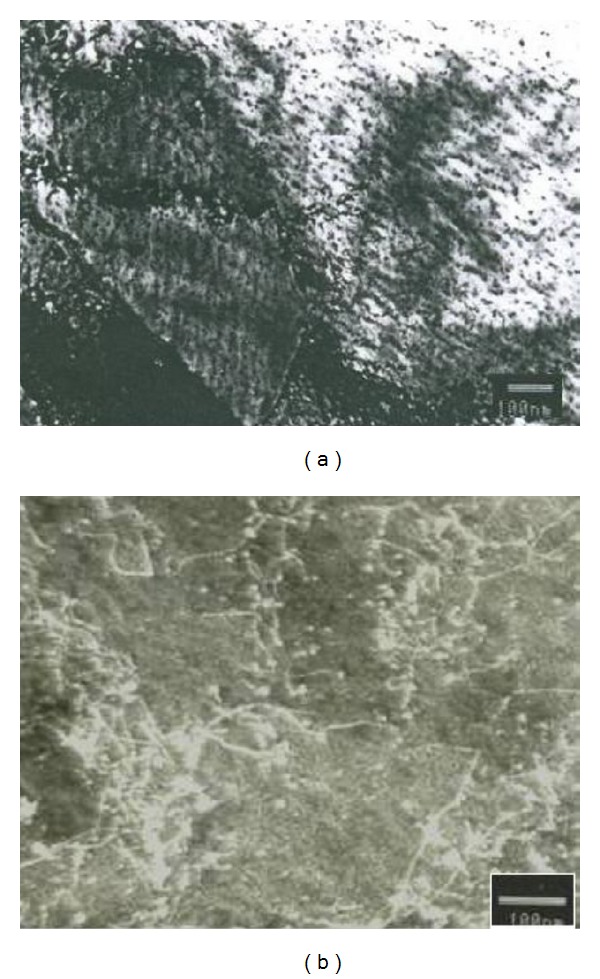
Niobium precipitates in hot-rolled strip (2). (a) Deformation induced series of niobium precipitates. (b) Interaction of precipitates and dislocations.

The occurrence was associated with the influence of niobium precipitates on flow of deformation [2]. In the final thermomechanical treatment, comes the extraction of deformation-induced precipitates in the form of densely complex carbonitride series arranged in grains and across multiple grains. Transmission electron microscopy, Figure 2, found the series of Nb(CN) precipitates and their interaction with dislocations [2].

By the present knowledge about the rheological behavior of some steels, behavior of niobium microalloyed steels in the beginning of plastic deformation can be related to pinning of dislocations movement on the series of precipitates.

**Figure 3:**
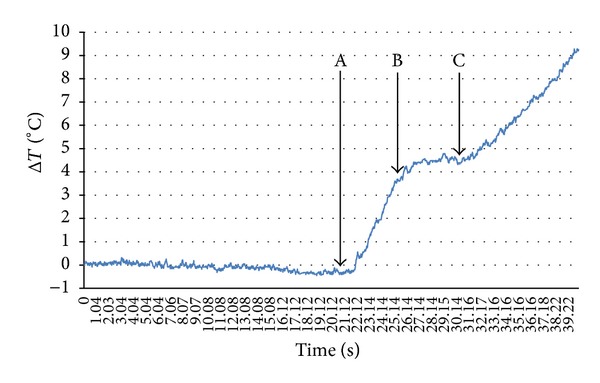
Changes of maximum temperature during the elastic and at the beginning of plastic deformation.

Results of parallel thermographic measurement clearly show the change of temperature in that area, Figure 3.

From Figure 3, it is visible that, in the area of elastic deformation, temperature drops. Minimum measured temperature drop was −0.41°C. By comparing the load-elongation curve of the sample, Figure 1, the point of proportionality corresponds to the point with the highest temperature drop, Figure 3, point A. On the low carbon steel, it was recorded maximum temperature drop of Δ*T* = −0.35°C under the same testing conditions [10]. Similar studies carried out on austenitic steel [11] showed a drop in temperature of Δ*T* = −0.18°C. Measured drop in temperature during the elastic deformation of the materials is explained by thermoelastic effect [10, 11].

**Figure 4:**
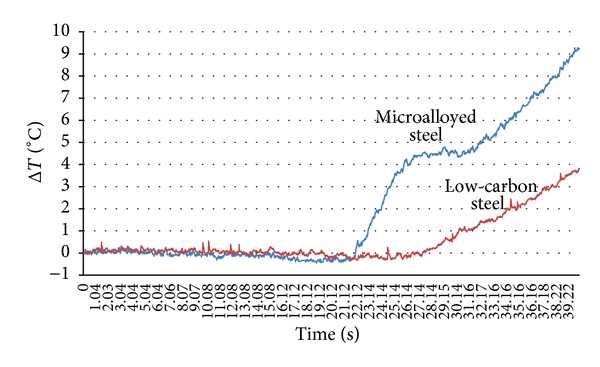
Change in maximum temperatures during the elastic and at the beginning of plastic deformation of low-carbon and niobium microalloyed steel.

After reaching the proportionality limit, point A, a specific behavior of niobium microalloyed steel is observed. First, the temperature rapidly increases to 4.5°C and then remains approximately constant for a brief period. This phenomenon was not recorded at low-carbon steel, Figure 4. The beginning of plastic flow of material starts later and has a continuous increase.

**Figure 5:**
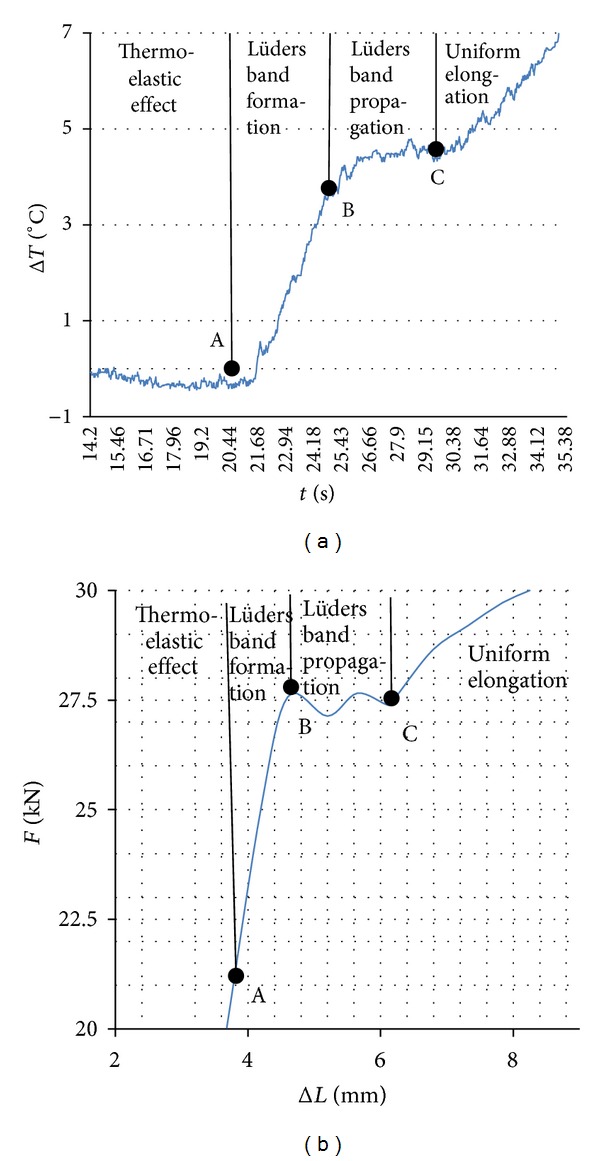
Zones of formation and propagation of Lüders band. (a) Temporal variations of temperature of niobium microalloyed steel. (b) Load-elongation curve.

The area in which a specific increase in temperature occurs at microalloyed steel, from point A to C in Figure 3, corresponds to an area of its rheological behavior at the beginning of plastic flow of material. The comparison was made in Figure 5.

According to Figure 5, the deformation process can be divided into four zones. In the first zone, from 0 to A, thermoelastic effect is clearly visible. In the second zone, from A to B, the temperature sharply increases, and in the third zone from B to C, it remains constant for a specific period. After C, temperature increases again.

**Figure 6:**
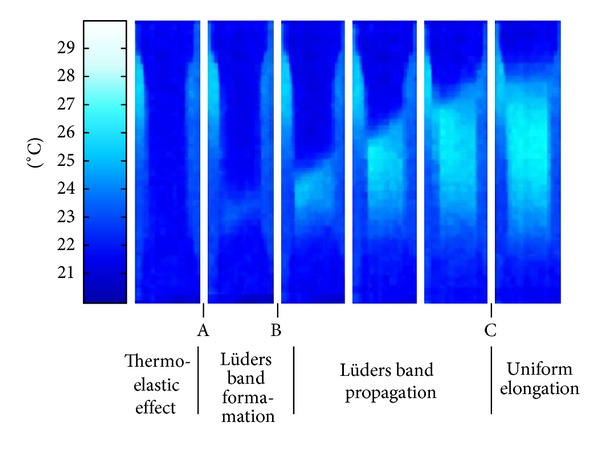
Thermograms of temperature distribution in the deformation zone.

In order to explain the observed changes in temperature, an analysis of thermovision tests was done, and in the above mentioned points, thermographic views of the sample were captured, Figure 6. On the thermograms, it was recorded a temperature distribution in the sample, respectively, in the zone of deformation.

Start of plastic flow of material is associated with a specific increase in temperature. The increase in temperature starts in point A, after reaching the proportionality limit. At the same time, both elastic and plastic deformations take place. Elastic deformation occurs by acting of normal stresses. For plastic deformation, tangential stresses acting at the angle of 45° to the direction of the force, or angles close to this value, are required. Accumulation of dislocations on the series of niobium precipitates postpones the beginning of plastic deformation. Stress increase occurs as temperatures increases and it is shown in Figure 5. At this point, in the part of the crystal structure with the highest potential energy, a front of deformed and nondeformed structure is formed, respectively, Lüders band, Figure 6. It is fully formed in point B. At that point, the critical stresses achieved are sufficient, so dislocations have a sufficient energy to overcome the obstacles. They start, point B, and there is a sudden drop in stress in the material one which the stress-strain diagram manifested as lower yield point (*R*_eL_). In Figure 5, we can see that the temperature does not rise. Elastic strain on one side of Lüders band gradually turns into a complex three axial stresses, which has the consequence that moves line away from itself. Deformation that takes place from the upper yield points up until the beginning of a uniform increases in force and elongation, from point B to C; it is not homogeneous, but it moves along the sample in successive steps until the whole length of the sample reaches values *R*_eH_. Maximum temperature is on Lüders band and it does not change. In this way, gradually the entire sample is deformed to a certain amount of deformation. Figure 6 shows that up to the point C, Lüders band propagates through the entire length of the sample. Once the entire sample is deformed for that specific amount of deformation and Lüders band propagated through the entire length of the sample, a homogenous plastic deformation of the sample starts.

## 4. Conclusion

Research has shown that when stretching out the samples in the cold state at rate of stretching of 15 mm/s, the steel microalloyed with 0.048% niobium after reaching the proportional limit has a complex rheological behavior, which has not been obtained from the same steel that is not microalloyed with niobium.

Low carbon steel, which has a clearly defined yield point, when stretching of the sample, after reaching *R*_eH_, forms a deformation zone that spreads through the sample. At the same steel microalloyed with niobium, material behavior during tensile testing can be divided into 4 zones. In the first zone, during the deformation, comes the decrease in temperature. It clearly pronounced the thermoelastic effect. In the second zone, because of obstructing the movement of dislocations on series of niobium precipitates, the stress grows and Lüders band is formed. In the third zone, it propagates through the length of the test sample. After that, in the formed deformation zone, the plastic deformation takes place.

Further research on the changes in the distribution of niobium precipitates will show their role in the formation of the deformation zone at stretching.

## Figures and Tables

**Table 1:** Chemical composition of steels [2].

Element	C, %	Mn, %	Si, %	P, %	S, %	Al, %	Nb, %	N, %
Niobium microalloyed steel	0,12	0,78	0,18	0,011	0,018	0,020	0,048	0,008
Low carbon steel	0,13	0,77	0,18	0,010	0,019	0,020	—	—
